# Applying the standardized infection ratio for reporting surgical site infections in Australian healthcare facilities

**DOI:** 10.1017/ash.2023.478

**Published:** 2023-11-16

**Authors:** Stephanie K. Tanamas, Lyn-Li Lim, Ann L. Bull, Michael J. Malloy, Allen C. Cheng, Leon J. Worth

**Affiliations:** 1 Victorian Healthcare Associated Infection Surveillance System (VICNISS) Coordinating Centre, Melbourne, VIC, Australia; 2 Department of Infectious Diseases, University of Melbourne at the Peter Doherty Institute for Infection and Immunity, Melbourne, VIC, Australia; 3 Centre for Epidemiology and Biostatistics, Melbourne School of Population and Global Health, The University of Melbourne, Melbourne, VIC, Australia; 4 Department of Infectious Diseases, Alfred Hospital and Central Clinical School and School of Public Health and Preventive Medicine, Monash University, Melbourne, VIC, Australia; 5 Monash Infectious Diseases, Monash Health and School of Clinical Sciences, Monash University, Melbourne, VIC, Australia; 6 National Centre for Antimicrobial Stewardship, Department of Infectious Diseases, The University of Melbourne, Melbourne, VIC, Australia; 7 Sir Peter MacCallum Department of Oncology, University of Melbourne Cancer & Department of Infectious Diseases, Peter MacCallum Cancer Centre, Melbourne, VIC, Australia; 8 Department of Medicine, The University of Melbourne, Melbourne, VIC, Australia

## Abstract

**Objective:**

We explored the utility of the standardized infection ratio (SIR) for surgical site infection (SSI) reporting in an Australian jurisdiction.

**Design:**

Retrospective chart review.

**Setting:**

Statewide SSI surveillance data from 2013 to 2019.

**Patients:**

Individuals who had cardiac bypass surgery (CABG), colorectal surgery (COLO), cesarean section (CSEC), hip prosthesis (HPRO), or knee prosthesis (KPRO) procedures.

**Methods:**

The SIR was calculated by dividing the number of observed infections by the number of predicted infections as determined using the National Healthcare Safety Network procedure-specific risk models. In line with a minimum precision criterion, an SIR was not calculated if the number of predicted infections was <1.

**Results:**

A SIR >0 (≥1 observed SSI, predicted number of SSI ≥1, no missing covariates) could be calculated for a median of 89.3% of reporting quarters for CABG, 75.0% for COLO, 69.0% for CSEC, 0% for HPRO, and 7.1% for KPRO. In total, 80.6% of the reporting quarters, when the SIR was not calculated, were due to no observed infections or predicted infections <1, and 19.4% were due to missing covariates alone. Within hospitals, the median percentage of quarters during which zero infections were observed was 8.9% for CABG, 20.0% for COLO, 25.4% for CSEC, 67.3% for HPRO, and 71.4% for KPRO.

**Conclusions:**

Calculating an SIR for SSIs is challenging for hospitals in our regional network, primarily because of low event numbers and many facilities with predicted infections <1. Our SSI reporting will continue to use risk-indexed rates, in tandem with SIR values when predicted number of SSI ≥1.

## Introduction

Surveillance and reporting of surgical site infections have been demonstrated to reduce the burden of healthcare-associated infections.^1,2^ Monitoring ensures timely identification of increases in infection rates and enables evaluation of quality improvement activities. The Victorian Healthcare Associated Infection Surveillance System (VICNISS) Coordinating Centre provides a statewide program supporting hospital-level monitoring of surgical site infections, in accordance with methods employed by the National Healthcare Safety Network (NHSN).^3^ Data collated by the VICNISS Coordinating Centre facilitate identification of changes in infection etiology, evaluation of time trends, and benchmarking activities.

To date, the VICNISS network has utilized the National Nosocomial Infections Surveillance (NNIS) risk index^4^ to report risk-stratified surgical site infection rates. The performance of the risk index when applied to VICNISS data was found to be adequate for most procedures,^5^ though only a very weak correlation with infection rates was found for cardiac bypass surgery (CABG), and an alternative risk prediction score was thus explored.^6^ Over the last decade, the standardized infection ratio (SIR) has been increasingly applied by other programs as an enhanced method for risk adjustment.^7^ Standardization is a method used to control for differences between populations where their demographics or other characteristics may confound their comparison. Indirect standardization compares the number of observed events against the number of events that are expected and is preferred when event rates are low. The SIR is an indirect method of standardization that has been the primary method used for risk-adjusted surgical site infection reporting in the US since 2009.^8,9^ Here, expected rates are estimated from facility- and patient-level variables. There are several advantages to the SIR compared to traditional risk-stratified rates. Risk-stratified rates allow for comparison within strata only and do not present an overall procedure-specific performance metric for a hospital. With the SIR, one summary measure of a hospital’s performance can be calculated per procedure. The SIR adjusts for procedure-specific covariates where the risk index stratifies by the same three covariates for all procedures (with exceptions such as the use of a laparoscope in colorectal surgery). For some procedures, the factors that make up the NNIS risk index are not associated with or of equal importance in the extent to which they affect infection risk.^5,7^

Australian healthcare facilities and healthcare-associated infection surveillance programs do not currently report the SIR, and feasibility has not been formally evaluated with respect to surgical site infection surveillance in our region. The objective of this study was to evaluate the utility of the NHSN procedure-specific SIR for surgical site infection reporting in an Australian jurisdiction, including benefits and limitations. We specifically sought to calculate SIR values for infection events following 5 surgical procedures in hospitals within our network, to compare SIR values with risk-stratified rates, and to determine factors contributing to inability to calculate the SIR.

## Methods

### Study population

There were over 6.5 million people living in Victoria according to the 2021 Census.^10^ The hospital system in Victoria comprises over 200 public and private hospitals.^11^ Public hospitals provide free care to all Australian citizens and permanent residents. They are more easily accessible, especially in rural areas, and tend to be better equipped to handle more complex cases. The fee for patients in private hospitals is covered by a combination of Medicare (publicly funded universal health care insurance scheme), private health insurance, and patients’ own funds.

### Surveillance system

Submission of surgical site infection surveillance data to the VICNISS Coordinating Centre has been a requirement since 2002 for all eligible Victorian public hospitals. Initially, this included all public hospitals performing CABG and a minimum number of hip and knee replacements annually (HPRO and KPRO, respectively). Some years later, the requirement was expanded to reporting infections following cesarean sections (CSEC) for hospitals providing healthcare for women, and colorectal surgery (COLO) for hospitals performing more than 50 procedures annually. Private hospitals began voluntarily submitting surgical site infection surveillance data in 2009.

### Dataset for analysis

Surgical site infection surveillance data from January 2013 to December 2019 for CABG, COLO, CSEC, HPRO, and KPRO procedures were extracted from the VICNISS database. These procedure groups were selected to represent a range of procedures (clean and non-clean), elective, and emergency settings. Patient-level covariates reported to VICNISS can be found in the Supplementary Material (Forms A and B). Available hospital-level covariates include hospital type (public/private), acute bed numbers, and geographical location.

Procedures performed for patients aged <18 years and hospitals reporting <50 procedures annually between 2013 and 2019 were excluded from analysis. Data from 2020 to 2022 were excluded as hospitals were given the option to reduce or pause participation in VICNISS surgical site infection surveillance during this period due to the COVID-19 pandemic.

### Standardized infection ratio

The SIR was calculated by dividing the observed number of infections by the predicted number of infections, with the latter determined using National Healthcare Safety Network (NSHN) procedure-specific risk models.^7^ Medical school affiliation was omitted from the COLO risk model. Unlike US facilities, all Victorian healthcare facilities performing >50 colorectal procedures per year are associated with an academic center, meaning that use of this metric to classify healthcare facilities would not contribute meaningfully to quantitative risk assessment. No imputation was performed for missing covariates, except for acute bed numbers where the most recent non-missing bed number for that hospital was used.

In accordance with recommendations of the Centers for Disease Control and Prevention for a minimum precision criterion, an SIR was not calculated if the number of predicted infections was <1.^12^

### Risk-stratified surgical site infection rate

Risk-stratified surgical site infection rates for each studied operative procedure were calculated based on the NNIS risk index.^4^ One point was allocated for each of the following: (i) American Society of Anesthesiologists (ASA) score ≥3; (ii) a wound class of either contaminated or dirty; and (iii) operation lasting more than *t* hours, where *t* is the approximate 75th percentile of the duration of surgery for that operative procedure.^13^ A modified risk index was used for COLO procedures incorporating the use of a laparoscope, subtracting 1 from the patient’s risk index if surgery was performed laparoscopically.^14^

### Statistical analysis

The frequency of 0 or 1 reported surgical site infections and calculation of an SIR >0 was summarized by procedure and time epoch. Different time epochs (quarter, half-year, and year) were explored as alternative levels of data aggregation with the goal of reducing the frequency of 0 infections, and thus a 0 SIR, in the reporting period.

Spearman’s rank correlation was used to evaluate the relationship between the SIR and risk-stratified infection rates while allowing for the skewed distribution of the data.

### Ethics review

The VICNISS Coordinating Centre, on behalf of the Victorian Department of Health and Human Services, collects surveillance data to support healthcare quality improvement activities. For the current study, de-identified data were retrospectively analyzed for the purpose of informing future reporting frameworks, consistent with national guidelines for quality assurance activities.^15^ As such, ethics review was not required.

## Results

The total number of procedures, surgical site infections, and reporting hospitals is summarized in Table 1, overall and by hospital type (public/private).

**Table 1.** Number of procedures, surgical site infections, and hospitals reporting on each procedure, by surgical procedure and hospital type

	Number of hospitals			Number of procedures			Number of infections		
Procedure	Total	Public	Private	Total	Public	Private	Total	Public	Private
Cardiac bypass surgery	10	6	4	16,583	13,224	3,359	582	520	62
Colorectal surgery	23	22	1	10,456	10,163	293	740	734	6
Cesarean section	28	25	3	81,600	78,875	2,725	728	721	7
Hip replacement	44	29	15	42,956	31,320	11,636	515	406	109
Knee replacement	44	29	15	37,544	23,230	14,314	350	229	121

### SIR calculation by hospital

**Figure 1.**
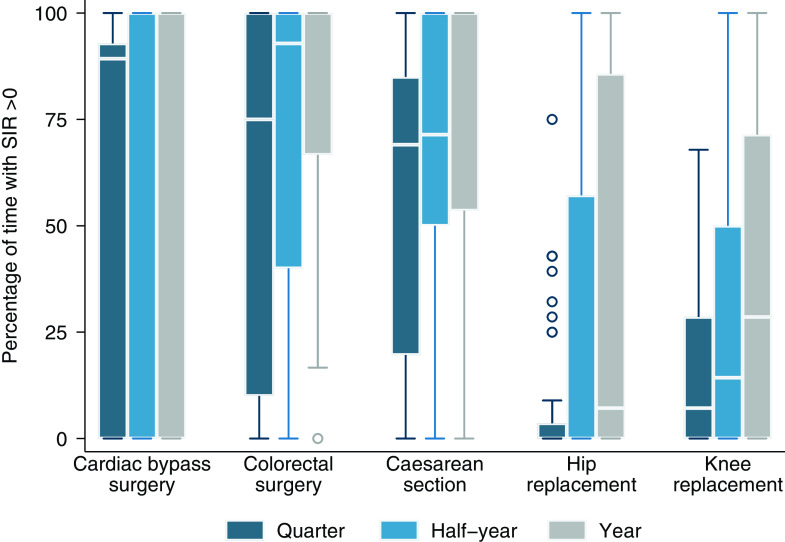
Percentage of time when hospitals had a standardized infection ratio >0, by procedure and time interval. The horizontal line through the box indicates the median, the vertical length of the box represents the interquartile range (IQR), the whiskers span all data points within 1.5 IQR + 25th percentile and 1.5 IQR + 75th percentile, and the open circles denote outliers.

The percentage of time for which hospitals were able to calculate an SIR >0 for each procedure is summarized in Figure 1. An SIR >0 was calculated when at least 1 infection was observed, there were no missing covariates, and the predicted number of infections was ≥1. These conditions were met with high frequency for CABG, COLO, and CSEC, but infrequently for HPRO and KPRO even when infection numbers were aggregated by year (Supplementary Table S1).

For several hospitals, an SIR >0 was never able to be calculated (Supplementary Table S2). Absence of observed infections was the most common cause for this when data were aggregated by quarter while missing covariates became the dominant cause when data were aggregated by year (Supplementary Table S3). When an SIR >0 was calculated, 62% (349 out of 561) of quarterly SIR values, 47% (208 out of 444) of half-year SIR values, and 30% (95 out of 321) of yearly SIR values were derived from a single observed infection during the surveillance period.

### SIR and risk-stratified infection rates

**Figure 2.**
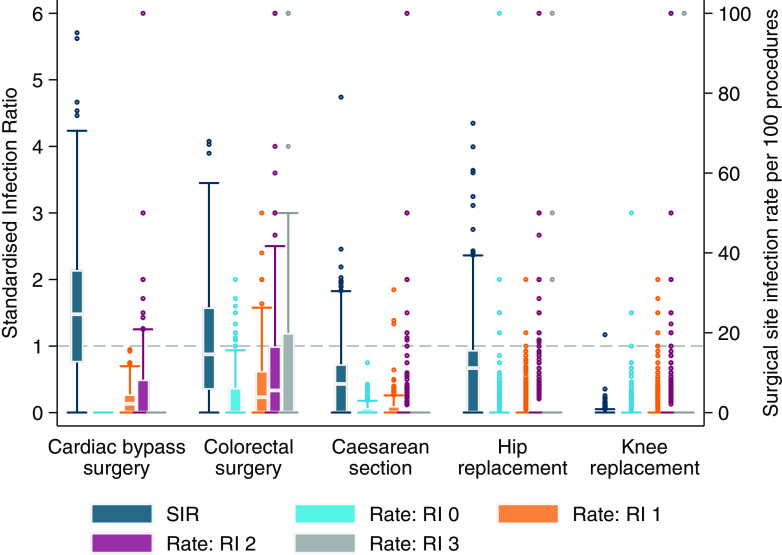
Standardized infection ratio (SIR) and risk-stratified surgical site infection rates calculated for each quarter by procedure and risk index (RI). Horizontal dashed line indicates SIR = 1. The horizontal line through the box indicates the median, the vertical length of the box represents the interquartile range (IQR), the whiskers span all data points within 1.5 IQR + 25th percentile and 1.5 IQR + 75th percentile, and the open circles denote outliers.

The quarterly SIR and risk-stratified infection rates for individual hospitals are summarized in Figure 2 and Supplementary Table S4 and the correlation between quarterly SIR and risk-stratified rates is summarized in Table 2. The median SIR was <1 for all procedures except for CABG. Notably, quarterly hospital-level SIR was <1 for KPRO for all but 1 quarter at 1 hospital. A wider range of infection rates was found for COLO procedures when compared to the other procedures. There were few infections reported in risk index category 3: 40 following COLO, 4 following HPRO, 1 following KPRO, and none following CABG or CSEC. There were no infections following CABG reported in risk index 0.

**Table 2.** Correlation between standardized infection ratio and risk-stratified surgical site infection rates calculated by reporting quarter

	Risk Index				
Procedure	0	1	2	3	−1
Cardiac bypass surgery	–	0.89	0.87	–	N/A
Colorectal surgery	0.87	0.89	0.93	0.98	0.99
Cesarean section	0.73	0.84	0.95	–	N/A
Hip replacement	0.93	0.89	0.88	–^a^	N/A
Knee replacement	0.91	0.87	0.97	–^a^	N/A

Note. SIR, standardized infection ratio; N/A, not applicable.

a SIR is not calculated as number of predicted surgical site infections <1.

### Percentage of time when hospitals observed no more than 1 surgical site infection

**Figure 3.**
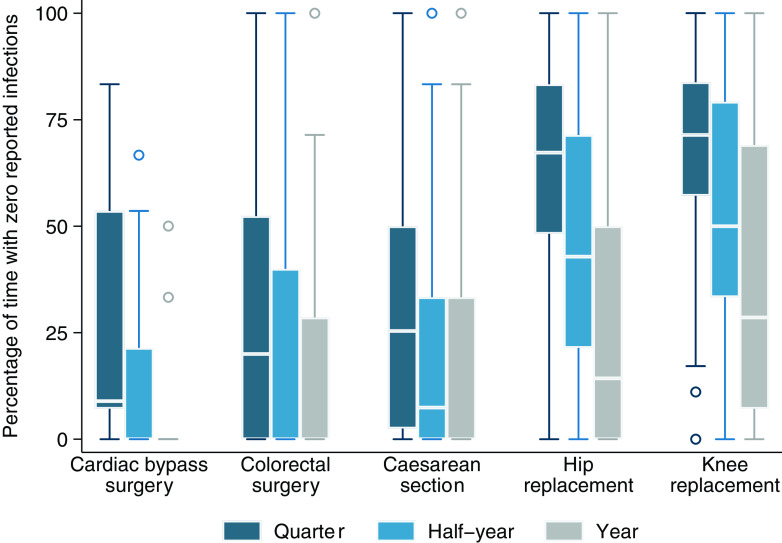
Percentage of time when hospitals reported 0 infections, by procedure and time interval. The horizontal line through the box indicates the median, the vertical length of the box represents the interquartile range (IQR), the whiskers span all data points within 1.5 IQR + 25th percentile and 1.5 IQR + 75th percentile, and the open circles denote outliers.

**Figure 4.**
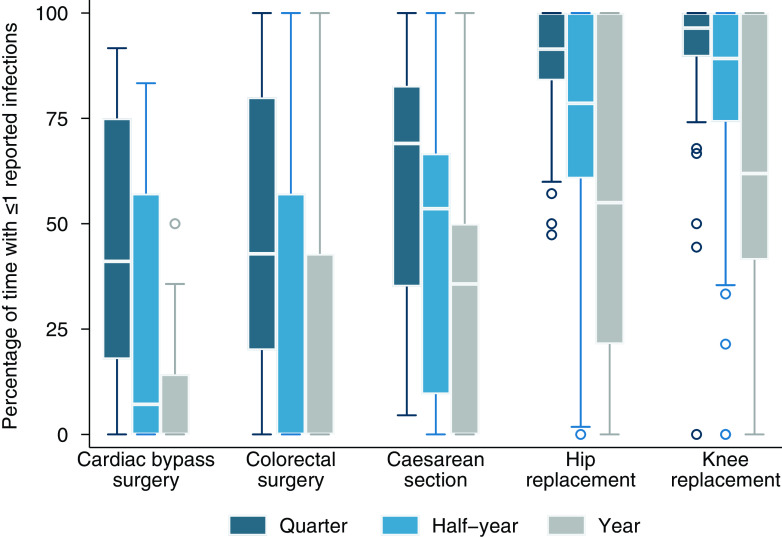
Percentage of time when hospitals reported 1 infection or less, by procedure and time interval. The horizontal line through the box indicates the median, the vertical length of the box represents the interquartile range (IQR), the whiskers span all data points within 1.5 IQR + 25th percentile and 1.5 IQR + 75th percentile, and the open circles denote outliers.

Within hospitals, the percentage of time between 2013 and 2019 during which 0 infections were observed is summarized in Figure 3 and Supplementary Table S5. The occurrence of 0 infections was considerably lower when the period of observation was increased from 3 months to six or 12 months, most notably for CABG. Even when infections were observed, the number of events remained very small and often no more than 1 per quarter (Figure 4 and Supplementary Table S6e.

### Missing covariates

Evaluation of missing covariate data revealed “acute bed number,” which was missing for 27.2% of patients, to be the most frequently missing covariate (Supplementary Table S7). Other covariates were missing for ≤3% of patients. The number and percentage of hospitals and patients with missing bed number is tabulated in Supplementary Table S8.

## Discussion

Our study is the first to explore the merit of the SIR for reporting healthcare-associated infections in an Australian jurisdiction. Most notably, we identified the difficulty in applying the SIR method for routine surgical site infection reporting when infection numbers are low.

The 5 procedures examined in this study varied widely in their infection frequency at the hospital level, and the value of the SIR for reporting surgical site infections varied accordingly. Consider that 582 CABG infections were reported between 2013 and 2019 across 10 hospitals, compared to 515 HPRO infections and 350 KPRO infections reported across 44 hospitals within our network. CABG and COLO infections occur at sufficiently high frequencies that routine SIR calculation could be informative, at least for some hospitals. The utility of the SIR for routine reporting of CSEC infections is limited since infection numbers per hospital tended to be lower than for CABG and COLO, and even more limited for HPRO and KPRO where infections were a relatively rare event.

For at least half of the hospitals reporting on CAGB and COLO, there was no more than 1 infection reported per quarter almost half of the time. This proportion was dramatically higher for HPRO and KPRO and remained above 50% even when the number of infections was summed over 12-month periods. With 0 observed infections, no risk adjustment is necessary, and with only 1 observed infection, the SIR would at worst indicate that a hospital’s infection numbers are as expected relative to a reference standard, given that the denominator (number of predicted infections) cannot be less than one.^12^ Under this scenario, hospitals can never perform worse than expected based on the reference standard. This is particularly evident in Figure 2 where the SIR for KPRO showed that all hospitals performed better than expected almost 100% of the time, an unsurprising observation given that more than a quarter of hospitals never report more than 1 infection in a year.

A potential approach to strengthen analysis of datasets with a small number of events is to aggregate data. We modeled this using quarterly, half-yearly, and yearly epochs. Quarterly SIR calculation was explored as this would align with the frequency of reporting for other health metrics provided by VICNISS to the Victorian Department of Health. We found that application of the SIR method within quarterly time intervals was difficult as the number of infections was often very low, and data aggregation by at least half-year intervals was needed to reduce the frequency of 0 observed infections. Data aggregation could also be achieved by combining hospitals by service type (e.g. public/private, small/large, or by peer group^16^). However, we believe such aggregation may limit the interpretation and meaningfulness of data for clinical stakeholders and would limit the capacity to identify target areas for quality improvement. Utility of the SIR depends on the purpose for which the SIR is calculated. Aggregation by larger time intervals can be informative as a high-level performance indicator, such as in annual reports, but less useful as a metric for hospitals to use to identify and address local performance issues in a timely manner. Aggregation by hospital type may also be appropriate for high-level reporting but makes information difficult to interpret for individual hospitals.

The SIR method is limited by ecological fallacy in that hospital-level factors may not indicate patient-level risk. Others have cautioned that the SIR method may mathematically obscure hospital under-performance by including hospital-level covariates in the risk model.^17,18^ The SIR is easy to interpret as a simple ratio of the number of infections observed at a hospital relative to the number of expected infections insofar as an SIR <1 indicates better than expected performance and an SIR >1 indicates worse than expected performance. However, how the components of the SIR are derived may be less intuitive for stakeholders in hospitals, and for others less familiar with risk prediction models, than risk-stratified rates. The denominator of the SIR is the number of infections that is expected based on a reference standard. While the SIR method is routinely applied to reporting of healthcare-associated infections (HAIs) in the US,^8^ it is not routinely used in Australia and thus it may not be apparent to hospitals in the VICNISS network that the NHSN risk model’s reference standard is based on the average hospital in the US and not Australia. This, in combination with the low infection numbers observed at the hospital level in Victoria, suggests that the SIR does not provide an enhanced reporting metric when compared to traditional risk-stratified rates for surgical site infection reporting for Victorian healthcare facilities. We note that similar findings were reported with respect to central-line bloodstream infection surveillance in the US hospitals.^19^

Although the reporting of no infections by many hospitals posed the foremost barrier to calculation of the SIR in our dataset, predicted infections <1 and missing covariates were additional contributing factors limiting the capacity to calculate the SIR. Acute bed number was the most frequently missing covariate in our dataset over time, specifically for private hospitals. Looking ahead it is anticipated that more complete data for public hospitals will be accessible through the data custodian in the state of Victoria (Department of Health) but will likely remain a challenge for private hospitals. Improved data validation practices could reduce the number of missing covariates, although it is unlikely to fully address this limitation. Future work is warranted to explore whether recalibration of the NHSN model or development of a new risk model^20,21^ using the VICNISS hospital cohort as the reference standard could improve model performance. While this may enhance the accuracy of the number of predicted infections, the overarching issues of predicted infections being less than one and the availability of covariates within the VICNISS dataset will not be addressed by model refinement.

Calculation of quarterly SIRs for surgical site infections is challenging for hospitals in our regional network, given the small number of events, a substantial number of facilities with predicted infections <1, and missing data. We, therefore, propose for our surgical site infection reporting that: (i) traditional approaches (risk-indexed rates) be maintained, (ii) half-yearly or yearly SIR values be calculated in tandem with infection rates when predicted infections are ≥1, followed by an evaluation of how these data are used by individual healthcare facilities, and (iii) validation processes be enhanced for data submission.

## Supporting information

Tanamas et al. supplementary material 1 Tanamas et al. supplementary material

Tanamas et al. supplementary material 2 Tanamas et al. supplementary material

Tanamas et al. supplementary material 3 Tanamas et al. supplementary material

## Supplementary material

The supplementary material for this article can be found at https://doi.org/10.1017/ash.2023.478.
