# Supplementary material for: Applying the standardized infection ratio for reporting surgical site infections in Australian healthcare facilities
Source: Antimicrob Steward Healthc Epidemiol. 2023 Nov 16;3(1):e211. doi: 10.1017/ash.2023.478 (PMC10753515; doi:10.1017/ash.2023.478)
Supplement: Tanamas et al. supplementary material 1 — Tanamas et al. supplementary material [file S2732494X23004783sup001.docx]

# Supplementary Material

**Table S1.** **Percentage of time when hospitals had a non-zero standardized infection ratio, by procedure and time interval**

|  | Median (25^th^ percentile – 75^th^ percentile) percentage of time | | |
| --- | --- | --- | --- |
| Procedure | Quarter | Half-year | Year |
| Cardiac bypass surgery | 89.3 (0 – 92.9) | 100 (0 - 100) | 100 (0 - 100) |
| Colorectal surgery | 75.0 (10.0 - 100) | 92.9 (40.0 - 100) | 100 (66.7 - 100) |
| Caesarean section | 69.0 (19.6 – 85.0) | 71.4 (50.0 - 100) | 100 (53.6 - 100) |
| Hip replacement | 0 (0 – 3.6) | 0 (0 – 57.1) | 7.1 (0 – 85.7) |
| Knee replacement | 7.1 (0 – 28.6) | 14.3 (0 – 50.0) | 28.6 (0 – 71.4) |

**Table S2.** **Frequency** **of hospitals that were never able to calculate a non-zero standardized infection ratio, by procedure and time interval**

|  | Number (%) of hospitals | | |
| --- | --- | --- | --- |
| Procedure | Quarter | Half-year | Year |
| Cardiac bypass surgery | 4 (40.0) | 4 (40.0) | 4 (40.0) |
| Colorectal surgery | 5 (21.7) | 2 (8.7) | 2 (8.7) |
| Caesarean section | 6 (21.4) | 3 (10.7) | 2 (7.1) |
| Hip replacement | 32 (72.7) | 26 (59.1) | 22 (50.0) |
| Knee replacement | 18 (40.9) | 18 (40.9) | 18 (40.9) |

**Table S3. Frequency of missing or zero SIR by time interval and cause**

|  | Number (%) of time | | |
| --- | --- | --- | --- |
| Cause | Quarter | Half-year | Year |
| No observed infections | 343 (30.8) | 127 (29.1) | 50 (26.0) |
| Predicted infections <1 | 200 (18.0) | 66 (15.1) | 18 (9.4) |
| No observed infections and predicted infections <1 | 355 (31.9) | 82 (18.8) | 16 (8.3) |
| Missing covariates | 216 (19.4) | 162 (37.1) | 108 (56.3) |

**Table S4. Standardized infection ratio (SIR) and risk-stratified surgical site infection rates calculated for each quarter by procedure and risk index**

|  |  | Surgical site infection rate | | | | |
| --- | --- | --- | --- | --- | --- | --- |
| Procedure | SIR | Risk Index 0 | Risk Index 1 | Risk Index 2 | Risk Index 3 | Risk Index -1 |
| Cardiac bypass surgery | 1.48 (0.75 – 2.15) | - | 2.22 (0 – 4.65) | 0 (0 – 8.33) | - | N/A |
| Colorectal surgery | 0.88 (0.34 – 1.58) | 0 (0 – 6.25) | 3.85 (0 -10.53) | 5.56 (0 – 16.67) | 0 (0 - 20) | 0 (0 - 0) |
| Caesarean section | 0.43 (0 – 0.73) | 0 (0 – 1.18) | 0 (0 – 1.71) | 0 (0 - 0) | - | N/A |
| Hip replacement | 0.67 (0 – 0.95) | 0 (0 - 0) | 0 (0 - 0) | 0 (0 - 0) | 0 (0 - 0) | N/A |
| Knee replacement | 0 (0 – 0.02) | 0 (0 - 0) | 0 (0 - 0) | 0 (0 - 0) | 0 (0 - 0) | N/A |

Data is median and interquartile range.

**Table S5.** **Percentage of time when hospitals reported zero infections, by procedure and time interval**

|  | Median (25^th^ percentile – 75^th^ percentile) percentage of time epochs | | |
| --- | --- | --- | --- |
| Procedure | Quarter | Half-year | Year |
| Cardiac bypass surgery | 8.9 (7.1 – 53.6) | 0 (0 – 21.4) | 0 (0 - 0) |
| Colorectal surgery | 20.0 (0 – 52.4) | 0 (0 – 40.0) | 0 (0 – 28.6) |
| Caesarean section | 25.4 (2.5 – 50.0) | 7.4 (0 – 33.3) | 0 (0 – 33.3) |
| Hip replacement | 67.3 (48.2 – 83.3) | 42.9 (21.4 – 71.4) | 14.3 (0 – 50.0) |
| Knee replacement | 71.4 (57.1 – 83.8) | 50.0 (33.3 – 79.2) | 28.6 (7.1 – 69.0) |

**Table S6.** **Percentage of time when hospitals reported 1 infection or less, by procedure and time interval**

|  | Median (25^th^ percentile – 75^th^ percentile) percentage of time epochs | | |
| --- | --- | --- | --- |
| Procedure | Quarter | Half-year | Year |
| Cardiac bypass surgery | 41.1 (17.9 – 75.0) | 7.1 (0 – 57.1) | 0 (0 – 14.3) |
| Colorectal surgery | 42.9 (20.0 – 80.0) | 0 (0 – 57.1) | 0 (0 – 42.9) |
| Caesarean section | 69.0 (35.1 – 82.7) | 53.6 (9.5 – 66.7) | 35.7 (0 – 50.0) |
| Hip replacement | 91.4 (84.0 - 100) | 78.6 (60.7 - 100) | 55.0 (21.4 - 100) |
| Knee replacement | 96.4 (89.6 - 100) | 89.2 (74.2 - 100) | 61.9 (41.4 - 100) |

**Table S7. Number of patients and percentage of total with missing covariate data**

| Data field | Number | % | Procedure |
| --- | --- | --- | --- |
| Age | 17 | <0.1% | CABG, COLO, CSEC, HPRO, KPRO |
| Sex | 2 | <0.1% | CABG, KPRO |
| Body mass index^a^ | 2,497 | 3.06% | CSEC |
| ASA score | 48 | <0.1% | CABG, COLO, CSEC, HPRO, KPRO |
| Anesthesia used | 3,852 | 2.23% | COLO, CSEC, HPRO, KPRO |
| Procedure duration | 266 | 0.14% | CABG, COLO, CSEC, HPRO, KPRO |
| Acute bed numbers | 29,225 | 27.18% | CABG, COLO, HPRO, KPRO |
| Wound class | 20 | <0.1% | COLO, CSEC |
| Active labor in hospital | 6 | <0.1% | CSEC |
| Procedure was an emergency | 151 | 0.19% | CSEC |
| Laparoscope was used | 36 | 0.34% | COLO |
| Type of surgery | 158 | 0.20% | HPRO, KPRO |
| Trauma | 1,809 | 2.25% | HPRO, KPRO |

ASA = American Society of Anesthesiologists, CABG = cardiac bypass surgery, COLO = colorectal surgery, CSEC = caesarean section, HPRO = hip replacement, KPRO = knee replacement.

^a^ or height and weight

**Table S8. Number of patients and percentage of total with missing acute bed numbers***

| Procedure | Number (%) of hospitals | Number (%) of patients |
| --- | --- | --- |
| Cardiac bypass surgery | 4 (40.0) | 3,359 (20.3) |
| Colorectal surgery | 1 (4.3) | 293 (2.8) |
| Hip replacement | 15 (34.1) | 11,553 (26.9) |
| Knee replacement | 16 (36.4) | 14,020 (37.3) |

*Bed number is not a covariate in the risk prediction model for caesarean section surgical site infections.
