# Supplementary material for: Applying the standardized infection ratio for reporting surgical site infections in Australian healthcare facilities
Source: Antimicrob Steward Healthc Epidemiol. 2023 Nov 16;3(1):e211. doi: 10.1017/ash.2023.478 (PMC10753515; doi:10.1017/ash.2023.478)
Supplement: Tanamas et al. supplementary material 2 — Tanamas et al. supplementary material [file S2732494X23004783sup002.pdf]

Surgical Site Infection (Numerator)

THIS DATA MUST BE SUBMITTED ONLINE USING A VICNISS WEBFORM

Patient & Procedure Details	Hospital name:	
MRN (UR No.):	DOB:	Procedure Date:

Procedure Group:

☐ AAA	☐ CARD	☐ CHOL	☐ CSEC	☐ GAST	☐ HYST	☐ PACE	☐ VHSN
☐ APPY	☐ CBGB	☐ COLO	☐ FPOP	☐ HERN	☐ KPRO	☐ SB	
☐ BRST	☐ CBGC	☐ CRAN	☐ FUNS	☐ HPRO	☐ LAM	☐ VHYS	

Infection Details and Outcome	Date of Event (Infection Date):

Infection Detected: ☐ During admission ☐ Readmission (where procedure performed) ☐ Readmission to other facility ☐ Post discharge surveillance ☐ HITH

Infection Type: ☐ Superficial incisonal ☐ Deep incisional ☐ Organ/Space

If yes for Organ/Space infection, what was the Organ/Space Site:

☐ Arterial or venous infection (VASC)	☐ Myocarditis or pericarditis (CARD)
☐ Breast abcess or mastitis (BRST)	☐ Other infections of the lower respiratory tract (LUNG)
☐ Disc space infection (DISC)	☐ Osteomyelitis (BONE)
☐ Endocarditis (ENDO)	☐ Other infection of the male or femail reproductive tract (OREP)
☐ Endometritis (EMET)	☐ Periprosthetic joint infection (PJI)
☐ Gastrointestinal tract (GIT)	☐ Sinusitis (SINU)
☐ Intracranial infection (IC)	☐ Spinal abcess without meningitis (SA)
☐ Intraabdominal, not specified elsewhere (IAB)	☐ Urinary system infection (USI)
☐ Mediastinitis (MED)	☐ Vaginal cuff infection (VCUF)
☐ Meningitis or ventriculitis (MEN)	

Infection Present at time of surgery (PATOS): ☐ Yes ☐ No

CBGB only: Infection Site(s): ☐ Chest ☐ L Radial ☐ R Radial ☐ L Saphenous ☐ R Saphenous

Bilateral/2 Incision Procedures Only: Location of Infection: ☐ Left ☐ Right ☐ Other (specify):_________________________

Pathogen Details		
Pathogen Isolated: ☐ Yes ☐ No	Name of pathogen/s:<br>If listed below, complete antimicrobial susceptibility.	1.<br>2.<br>3.
Antimicrobial Susceptibility – see over page		

Surgical Site Infection (Numerator)

Antimicrobial Susceptibility - Record: S=Susceptible, I=Intermediate, R=Resistant*, or U=Unknown

Gram Positive Organisms	Cefoxitin	Ciprofloxacin	Cindamycin	Daptomycin	Doxycycline	Fucloxacillin	Gentamicin – high level test	Linezolid	Methicillin	Minocycline	Moxifloxacin	Oxacillin	Penicillin	Rifampicin	Teicoplanin	Tetracycline	Tigecycline	Trimethoprim/Sulfamethoxazole	Vancomycin
CNS (all species)																			
Enterococcus faecium																			
Enterococcus faecalis																			
Enterococcus spp.																			
Staphylococcus aureus																			

Antimicrobial Susceptibility - Record: S=Susceptible, I=Intermediate, R=Resistant*, or U=Unknown

Gram Negative Organisms	Amikacin	Amoxicillin-Clavulanic Acid	Ampicillin	Aztreonam	Cefalexin	Cefazolin	Cefepime	Cefotaxime	Ceftazidime	Ceftazidime/Avibactam	Ceftolozane/Tazobactam	Ceftriaxone	Ciprofloxacin	Colistin	Doripenem	Doxycycline	Ertapenem	Gentamicin	Imipenem	Imipenem/Relebactam	Levofloxacin	Meropenem	Meropenem/Vaborbactam	Minocycline	Moxifloxacin	Piperacillin-Tazobactam	Polyxin B	Tigecycline	Tobramycin	Trimethoprim-Sulfamethoxazole
Acinetobacter spp.																														
Escherichia coli																														
Enterobacter spp.																														
Klebsiella spp.																														
Pseudomonas aeruginosa																														
Serratia marcescens																														

Antimicrobial Susceptibility - Record:
S=Susceptible, R=Resistant, I=Intermediate, U=Unknown, or S-DD= Susceptible-dose dependent^

Candida Species *(specify name of organism if available)*	Anidulafungin	Caspofungin	Fluconazole^	Itraconazole^	Micafungin	Posaconazole	Voriconazole^
Candida_______________________________							
Candida_______________________________							

Note:

* Resistant is equivalent to non-susceptible
^ Susceptible-Dose Dependent (S-DD) is only applicable to Fluconazole, Itraconazole and Voriconazole
