# Supplementary material for: Applying the standardized infection ratio for reporting surgical site infections in Australian healthcare facilities
Source: Antimicrob Steward Healthc Epidemiol. 2023 Nov 16;3(1):e211. doi: 10.1017/ash.2023.478 (PMC10753515; doi:10.1017/ash.2023.478)
Supplement: Tanamas et al. supplementary material 3 — Tanamas et al. supplementary material [file S2732494X23004783sup003.pdf]

Surgical Procedure (Denominator)

THIS DATA MUST BE SUBMITTED ONLINE USING A VICNISS WEBFORM

Patient & Procedure Details	Hospital name:	MRN (UR No.):
Sex: ☐ M ☐ F ☐ O	DOB:	Admit Date:	Discharge Date:

Procedure Group: ☐AAA ☐BRST ☐CBGB ☐CHOL ☐CRAN ☐FPOP ☐GAST ☐HPRO ☐KPRO ☐PACE ☐VHYS ☐APPY ☐CARD ☐CBGC ☐COLO ☐CSEC ☐FUSN ☐HERN ☐HYST ☐LAM ☐SB ☐VSHN

ACHI codes/s: ________________ OR MBS Code/s:________________ OR Name of Procedure:________________

Procedure Date:	Start Time[1]: ☐NA	Finish Time[1]: ☐ NA	Surgeon (coded):
ASA Score: 1 2 3 4 5 NA	Wound Class: C CC CO D NA	Height (metres): ☐ NA	Weight: (kg): ☐ NA
General Anaesthesia: ☐Y ☐N	Emergency: ☐Y ☐N	Diabetes Mellitus: ☐Y ☐ N	Trauma: ☐ Y ☐ N
Closure Technique: ☐ Primary ☐ Non-primary	Laproscope: ☐ Y ☐ N	Robotic Assist: ☐ Y ☐ N	

HPRO/KPRO/BRST/HERN: ☐ Left ☐ Right ☐ Bilateral/2 Incisions[2] | CSEC: Duration of Labour (hours): __________ or ☐ No labour

HPRO/KPRO: (select one) ☐ Total ☐ Partial ☐ Resurfacing (HPRO only)
(select one) ☐ Primary ☐ Revision – if revision, was it associated with prior infection at index joint? ☐ Yes ☐ No ☐ NA

FUSN: Approach/Technique: ☐ Anterior ☐ Posterior ☐ Anterior & Posterior ☐ NA
Spinal Level: ☐ Atlas-axis ☐ Atlas-axis/Cervical ☐ Cervical ☐ Cervical/Dorsal/Dorsolumbar ☐ Dorsal/Dorsolumbar ☐ Lumbar/Lumbosacral ☐ NA

Surgical Antimicrobial Prophylaxis (SAP)

Prophylactic Antibiotic Administered: ☐ Yes ☐No ☐ NA

Antibiotic (Generic name)	Dose (mg)	Route	Time of Administration: Time Given	If exact time not given, tick box below	Antibiotic Continued: Beyond end of surgery	If yes, >24hrs post-op
1st Dose:						
				☐ >1hr prior to incision ☐ Within 1hr prior to incision ☐ On induction ☐ After Incision ☐ NA	☐Y ☐N	☐Y ☐N
				☐ >1hr prior to incision ☐ Within 1hr prior to incision ☐ On induction ☐ After Incision ☐ NA	☐Y ☐N	☐Y ☐N
				☐ >1hr prior to incision ☐ Within 1hr prior to incision ☐ On induction ☐ After Incision ☐ NA	☐Y ☐N	☐Y ☐N
Vancomycin[3] ☐ Y ☐ N				☐>120min prior to incision ☐ ≥15 to ≤120mins prior to incision ☐<15mins prior to incision ☐On induction ☐After Incision ☐NA	☐Y ☐N	☐Y ☐N
Clindamycin[3] ☐ Y ☐ N				☐>120min prior to incision ☐ ≥15 to ≤120mins prior to incision ☐On induction ☐After Incision ☐NA	☐Y ☐N	☐Y ☐N
Gentamycin[3] ☐ Y ☐ N				☐>120min prior to incision ☐ ≥15 to ≤120mins prior to incision ☐On induction ☐After Incision ☐NA	☐Y ☐N	☐Y ☐N
Metronidazole[3] ☐ Y ☐ N				☐>120min prior to incision ☐ ≥15 to ≤120mins prior to incision ☐On induction ☐After Incision ☐NA	☐Y ☐N	☐Y ☐N

2nd Dose: Did the surgery continue >4hrs after initial antibiotic pre-operative dose? ☐ Yes ☐ No
If yes, was the redosing of Cephazolin 4hrs from the initial preoperative dose? ☐ Yes ☐ No ☐ Not Required[4] ☐ NA

Other Antibiotic

In addition to SAP (listed above), was the patient receiving antibiotics prior to surgery (last 24hrs)? ☐ Yes ☐ No ☐ NA

Antibiotic (Generic name)	Dose (mg)	Route	Time of Administration: Time Given (last dose before surgery)	If exact time not available, tick a box below	
				☐ ≤1hr prior to incison ☐ >1- ≤4hrs prior to incision	☐ >4 - ≤12hrs prior to incision ☐ > 12 hr prior to incision
				☐ ≤1hr prior to incison ☐ >1- ≤4hrs prior to incision	☐ >4 - ≤12hrs prior to incision ☐ > 12 hr prior to incision

Outcome

Infection Detected: ☐Yes ☐No | Date of Event (Infection Date):

CBGB Only: Infection Site (s)[5]: ☐ Chest ☐ R Radial ☐ L Radial ☐ R saphenous ☐ L saphenous

1. If bilateral/2incisions HPRO/KPRO/BRST/HERN procedures are performed concurrently, duration of procedure should be inclusive of both procedures e.g. Left and right procedures. If performed sequentially and there are two procedure durations documented, submit the longest duration or if only one procedure duration divide by two and record half the entire duration.
2. Tick if two procedures (from the same procedure group) requiring 2 incisions were performed at the same time, e.g. left and right KPRO, umbilical and femoral HERN
3. Vancomycin, clindamycin, gentamicin & metronidazole – to accurately assess administration compliance the estimated start time intervals are specific to these antibiotics
4. Select 'Not Required' in settings where creatinine clearance <10ml/min (or eGFR<10) and antibiotic re-dosing is not required.
5. For CBGB procedures only - indicate <u>all</u> infection sites and complete a separate Surgical Site Infection (Numerator) Form for each infection.
